# Weighted gene coexpression correlation network analysis reveals a potential molecular regulatory mechanism of anthocyanin accumulation under different storage temperatures in ‘Friar’ plum

**DOI:** 10.1186/s12870-021-03354-2

**Published:** 2021-12-06

**Authors:** Xueling Li, Yudou Cheng, Meng Wang, Sujuan Cui, Junfeng Guan

**Affiliations:** 1 College of Life Science, Hebei Normal University, Shijiazhuang, Hebei Province 050024 People’s Republic of China; 2 Institute of Biotechnology and Food Science, Hebei Academy of Agriculture and Forestry Sciences, Shijiazhuang, Hebei Province 050051 People’s Republic of China; 3 Plant Genetic Engineering Center of Hebei Province, Shijiazhuang, Hebei Province 050051 People’s Republic of China; 4 Beijing Research Center for Agricultural Standards and Testing, Beijing Academy of Agricultural and Forestry Sciences, Beijing, People’s Republic of China

**Keywords:** Plum, WGCNA, Transcriptome, Anthocyanin, Storage

## Abstract

**Background:**

Flesh is prone to accumulate more anthocyanin in postharvest ‘Friar’ plum (*Prunus salicina* Lindl.) fruit stored at an intermediate temperature. However, little is known about the molecular mechanism of anthocyanin accumulation regulated by storage temperature in postharvest plum fruit.

**Results:**

To reveal the potential molecular regulation mechanism of anthocyanin accumulation in postharvest ‘Friar’ plum fruit stored at different temperatures (0 °C, 10 °C and 25 °C), the fruit quality, metabolite profile and transcriptome of its flesh were investigated. Compared to the plum fruit stored at 0 °C and 25 °C, the fruit stored at 10 °C showed lower fruit firmness after 14 days and reduced the soluble solids content after 21 days of storage. The metabolite analysis indicated that the fruit stored at 10 °C had higher contents of anthocyanins (pelargonidin-3-O-glucoside, cyanidin-3-O-glucoside, cyanidin-3-O-rutinoside and quercetin-3-O-rutinose), quercetin and sucrose in the flesh. According to the results of weighted gene coexpression correlation network analysis (WGCNA), the turquoise module was positively correlated with the content of anthocyanin components, and flavanone 3-hydroxylase (*F3H*) and chalcone synthase (*CHS*) were considered hub genes. Moreover, MYB family transcription factor APL (*APL*), MYB10 transcription factor (*MYB10*), ethylene-responsive transcription factor WIN1 (*WIN1*), basic leucine zipper 43-like (*bZIP43*) and transcription factor bHLH111-like isoform X2 (*bHLH111*) were closely related to these hub genes. Further qRT–PCR analysis verified that these transcription factors were specifically more highly expressed in plum flesh stored at 10 °C, and their expression profiles were significantly positively correlated with the structural genes of anthocyanin synthesis as well as the content of anthocyanin components. In addition, the sucrose biosynthesis-associated gene sucrose synthase (*SS*) was upregulated at 10 °C, which was also closely related to the anthocyanin content of plum fruit stored at 10 °C.

**Conclusions:**

The present results suggest that the transcription factors APL, MYB10, WIN1, bZIP43 and bHLH111 may participate in the accumulation of anthocyanin in ‘Friar’ plum flesh during intermediate storage temperatures by regulating the expression of anthocyanin biosynthetic structural genes. In addition, the *SS* gene may play a role in anthocyanin accumulation in plum flesh by regulating sucrose biosynthesis.

**Supplementary Information:**

The online version contains supplementary material available at 10.1186/s12870-021-03354-2.

## Background

Anthocyanin is a kind of water-soluble flavonoid that is derived from the branch of flavonoids, and it gives flowers and fruits various and graceful colours [1, 2]. As an antioxidant, anthocyanin can effectively remove free radicals such as reactive oxygen species (ROS) when plants suffer environmental stress, protecting plants from damage [3]. In addition, it has been shown that anthocyanin intake is beneficial to human prevention of cardiovascular diseases and cancer [4]; thus, anthocyanin has been widely studied recently.

The biosynthetic pathway of anthocyanins in higher plants is conserved, and anthocyanins are synthesized from phenylalanine catalysed by a series of enzymes. The enzyme-associated genes involved in anthocyanin synthesis are divided into early biosynthesis genes (EBGs) and late biosynthesis genes (LBGs) [5]. EBGs include chalcone synthase (CHS), chalcone isomerase (CHI), flavanone 3-hydroxylase (F3H) and flavonoid-3′-hydroxylase (F3’H), and they are common to different flavonoid synthesis branches [6, 7]. LBGs mainly include dihydroflavonol 4-reductase (DFR), leucoanthocyanidin dioxygenase/anthocyanin synthetase (LDOX/ANS) and UDP-glucose: flavonoid 3-O-glucosyltransferase (UFGT), and they contribute to the production of various anthocyanin components by catalysing flavanonol and its subsequent derivatives [8]. Anthocyanin biosynthesis-related genes are regulated by many transcription factors, among which MYB-bHLH-WD40 (MBW) has been widely studied. The MBW complex positively regulates the expression of structural genes by binding to *cis*-acting elements on the promoter regions of genes (such as *DFR*, *LDOX*/*ANS*, *UFGT*, etc.) and then facilitates the accumulation of anthocyanin in plants [7, 9–12]. In addition, transcription factors such as COP1 (CONSTITUTIVE PHOTOMORPHOGENIC 1), JAZ (JASMONATE ZIM-DOMAIN), NAC (NAM, ATAF1/2, CUC2), SPL (SQUAMOSA promoter-binding protein-like) and WRKY have been considered to regulate anthocyanin biosynthesis by interacting with the MBW complex [13–18].

Anthocyanin accumulation can be affected by light, temperature, hormones and mineral nutrition, and favourable low-temperature conditions are one of the important factors that induce the biosynthesis of anthocyanins [16, 19–24]. In grape fruit, cold treatment (4 °C) enhanced the expression levels of *VvF3H*, *VvPAL*, *VvCHS3*, *VvCHS2* and *VvLDOX* in the peel and subsequently led to anthocyanin accumulation [25]. Blood orange stored at 4 °C exhibited 3 times higher anthocyanin content in the flesh than that stored at 25 °C [20]. Compared with the 27 °C treatment, the apple fruit under a lower temperature (17 °C) showed a higher anthocyanin content and reddened quickly. Further study showed that the transcription factor *MdbHLH3* played a crucial role by interacting with *MdMYB1* to upregulate the expression of *MdDFR* and *MdUFGT* by binding their promotor regions [23]. In peach, the anthocyanin content in the flesh increased significantly when the fruit was stored at 16 °C, while the transcript levels of anthocyanin biosynthesis-associated genes were enhanced [26]. The flesh of ‘Friar’ plum fruit is prone to reddening when it is stored at 5–15 °C, and it was found that the main anthocyanin component is cyanidin-3-O-glucoside [27, 28]. However, the molecular regulation mechanism of the intermediate temperature causing flesh reddening in ‘Friar’ plum fruit is less well known. In the present study, flesh colouration in postharvest ‘Friar’ plum fruit stored at different temperatures (0 °C, 10 °C and 25 °C) was observed, and subsequently, metabolite profile and comparative transcriptome analysis in the flesh was performed. By constructing a coexpression network through WGCNA, hub genes and candidate regulatory transcription factors were identified. These findings provide new insights into the mechanism of intermediate temperature-induced anthocyanin accumulation in postharvest plum fruit.

## Results

### Effect of different storage temperatures on the fruit quality of ‘Friar’ plum

**Fig. 1:**
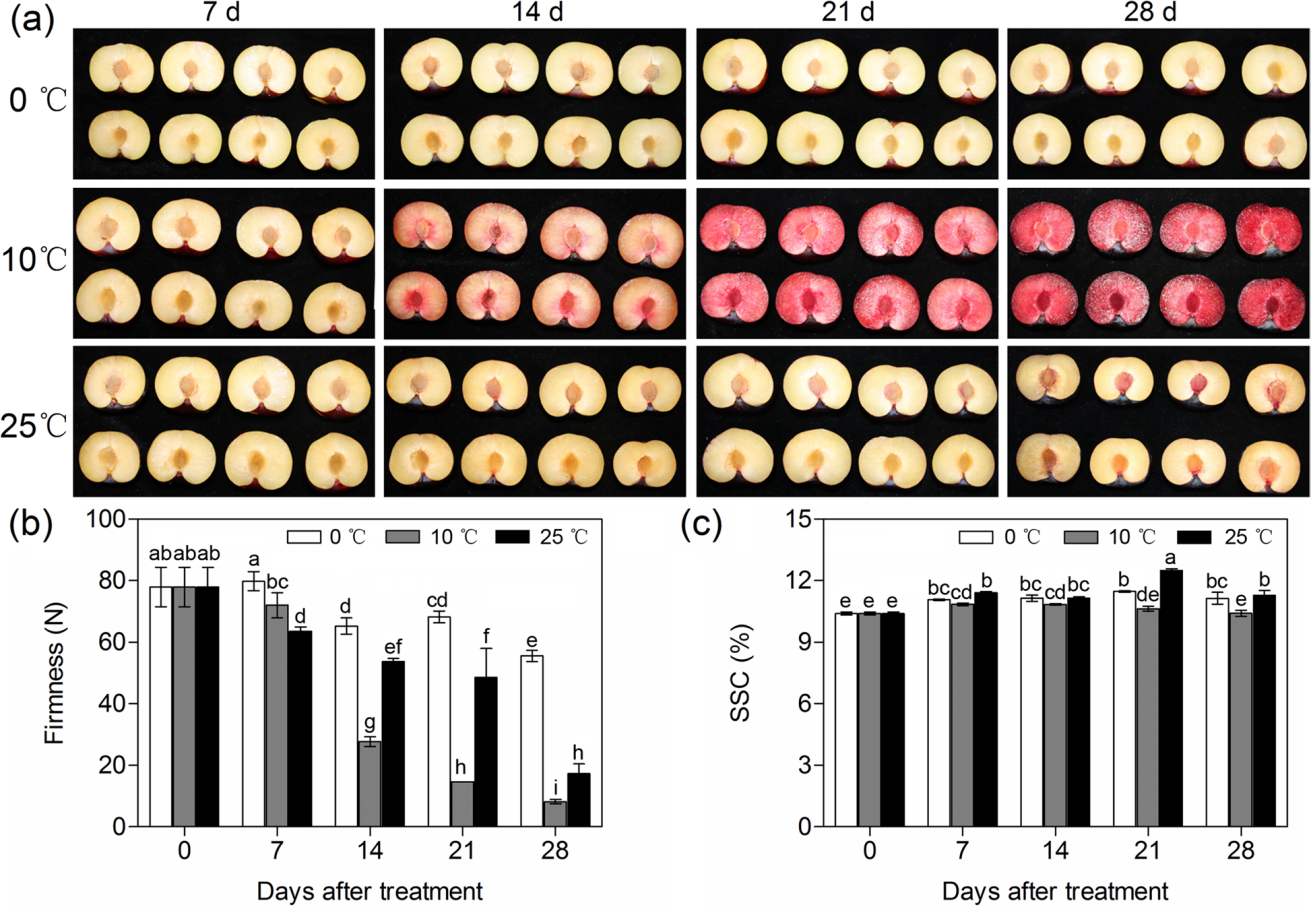
Quality changes of ‘Friar’ plum fruit under different storage temperatures. Flesh colour (**a**), firmness (**b**), and soluble solids content (SSC) (**c**). Different lowercase letters above the bars indicate significant differences (*P* < 0.05), which were obtained based on one-way ANOVA by LSD and DUNCAN tests

Within 28 days of storage, there were no obvious colour changes in the flesh of fruit stored at 0 °C and 25 °C, while the flesh of fruit stored at 10 °C turned red on the 14th day of storage (Fig. 1a). Among the three different storage temperatures, the firmness of the fruit stored at 10 °C decreased the fastest, while the fruit stored at 0 °C decreased the slowest (Fig. 1b). After 21 days of storage, the soluble solids content (SSC) in the fruit stored at 10 °C appeared significantly lower than that in the fruit stored at 0 °C and 25 °C (Fig. 1c).

### Identification of the anthocyanin and phenolic metabolite profile related to flesh reddening

**Fig. 2:**
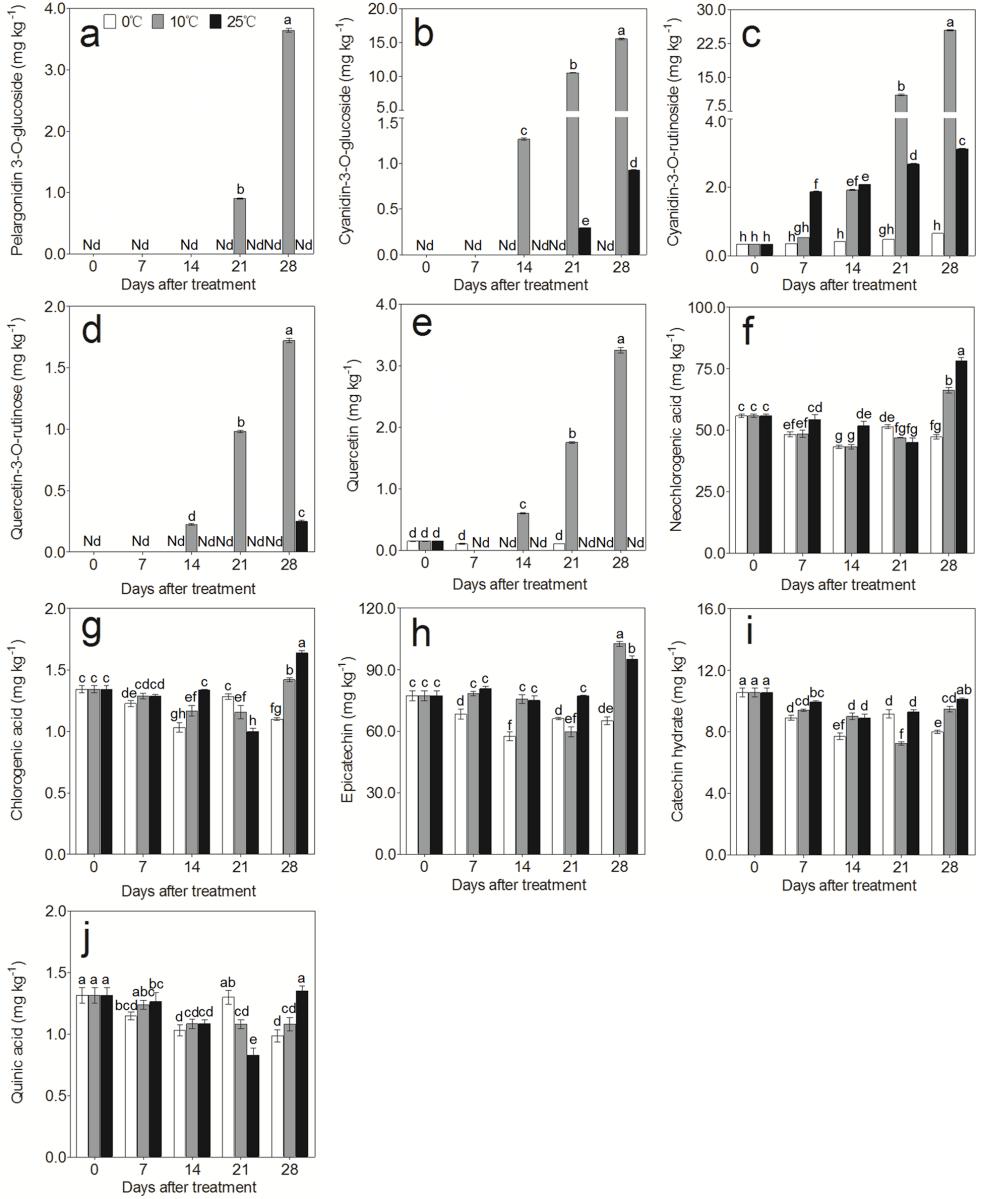
Changes in anthocyanin and polyphenolic components in ‘Friar’ plum flesh under different storage temperatures. **a** Pelargonidin-3-O-glucoside, **b** Cyanidin-3-O-glucoside, **c** cyanidin-3-O-rutinoside, **d** Quercetin-3-O-rutinose, **e** Quercetin, **f** Neochlorogenic acid, **g** Chlorogenic acid, **h** Epicatechin, **i** Catechin acid, and **j** Quinic acid. Different lowercase letters above the bars indicate significant differences (*P* < 0.05), which were obtained based on one-way ANOVA by LSD and DUNCAN tests. Nd stands for non-detectable

The UPLC–MS/MS results showed that the anthocyanins in the flesh of ‘Friar’ plum fruit mainly contained pelargonidin 3-O-glucoside, cyanidin-3-O-glucoside, cyanidin-3-O-rutinoside, and quercetin-3-O-rutinose, and their contents were much higher in fruit stored at 10 °C after 21 days than in those stored at 0 °C and 25 °C (Fig. 2a-d). Moreover, the contents of cyanidin-3-O-glucoside and cyanidin-3-O-rutinoside were much higher than pelargonidin 3-O-glucoside and quercetin-3-O-rutinose, indicating that cyanidin-3-O-glucoside and cyanidin-3-O-rutinoside were the main ingredients contributing to flesh reddening in ‘Friar’ plum fruit. In this work, polyphenolic components were also studied. Only the quercetin content in the flesh of fruit stored at 10 °C accumulated markedly, and the other polyphenolic components, such as neochlorogenic acid, chlorogenic acid, epicatechin, catechin hydrate, and quinic acid, were ubiquitous and less changed among all storage conditions (Fig. 2e-j), suggesting that quercetin was also related to the process of flesh reddening in ‘Friar’ plum fruit.

### Transcriptome analysis

**Fig. 3:**
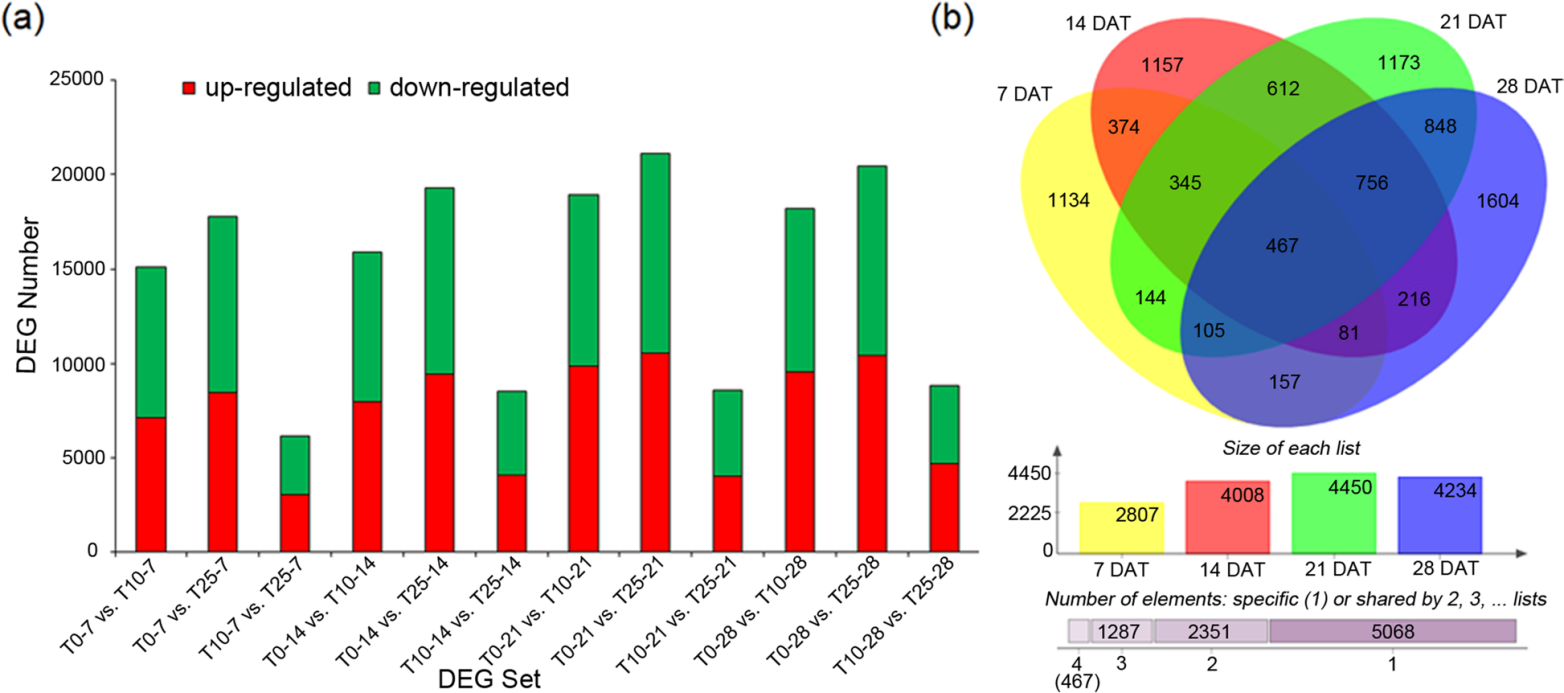
Summary of differentially expressed genes under different storage temperatures in ‘Friar’ plum. **a** Number of DEGs in different DEG sets. T0–7 vs. T10–7 represents the DEG set in which samples stored at 0 °C for 7 days versus samples stored at 10 °C for 7 days. **b** Venn diagram shows DEGs in both 10 °C vs. 0 °C and 10 °C vs. 25 °C at different storage time points. DAT represents days after treatment

To explore the regulatory mechanism of anthocyanin accumulation in the flesh of ‘Friar’ plum fruit, the samples at five detection time points (0, 7, 14, 21, 28 d) from the three different storage temperatures were used for deep RNA-seq analysis. After sequencing quality control, a total of 307.14 Gb of clean data were obtained, and the Q30 base percentage of each sample was not less than 91.47%. Then, the clean data were mapped to the European plum reference genome, with the mapping ratio varying from 84.98 to 93.00%. A total of 129,821 annotated genes were obtained. Differentially expressed genes (DEGs) were identified based on their expression levels in different samples, and functional annotation and enrichment analysis were performed. A total of 40,417 genes were differentially expressed under storage temperatures of 10 °C, 25 °C, and 0 °C (Fig. 3a). To screen the candidate genes related to anthocyanin biosynthesis, our study mainly focused on the DEGs at 10 °C vs. 0 °C and 10 °C vs. 25 °C. There were 2807, 4008, 4450, and 4234 genes that were differentially expressed on Days 7, 14, 21, and 28, respectively, at 10 °C vs. 0 °C and 10 °C vs. 25 °C (Fig. 3b). Among them, 467 genes were consistently differentially expressed (Fig. 3b).

### Construction of a WGCNA and coexpression network

**Fig. 4:**
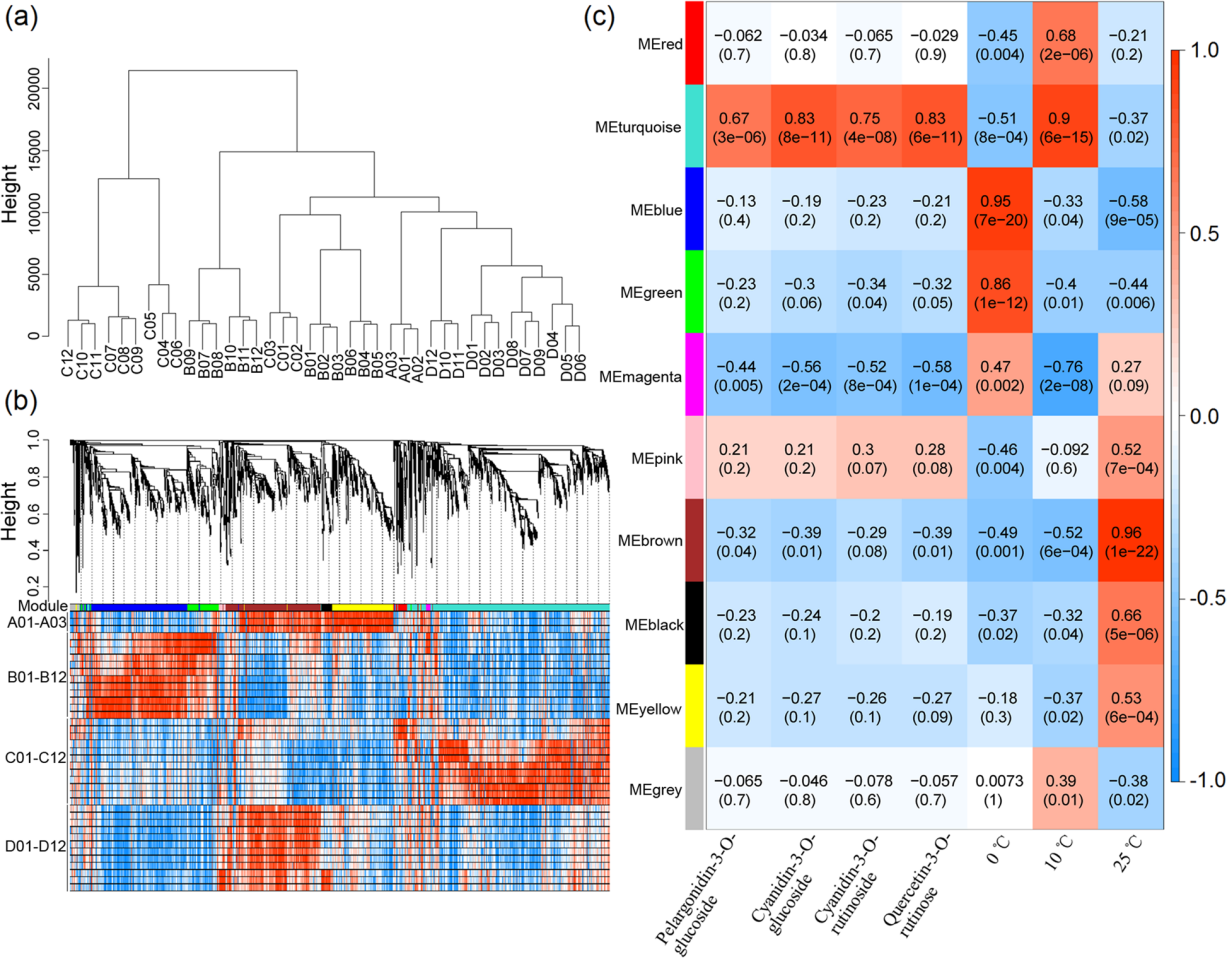
Weighted gene coexpression network analysis of ‘Friar’ plum fruit under different storage temperatures. **a** Sample clustering. A01–03 (initial sample); B01–03, C01–03, D01–03 (sample stored at 0 °C, 10 °C, and 25 °C for 7 days, respectively); B04–06, C04–06, D04–06 (sample stored at 0 °C, 10 °C, and 25 °C for 14 days), B07–09, C07–09, D07–09 (sample stored at 0 °C, 10 °C, and 25 °C for 21 days), B10–12, C10–12, D10–12 (sample stored at 0 °C, 10 °C, and 25 °C for 28 days). **b** Hierarchical clustering showing modules of coexpressed genes. **c** Module/trait correlations and corresponding *p* values. The right panel shows a colour scale for module/trait correlations from −1 to 1

To obtain hub genes related to anthocyanin accumulation, the relationships of DEGs, anthocyanin components and storage temperature for each sample were analysed by constructing a WGCNA (Fig. 4). Sample clustering showed that the three biological replicates of each treatment were very good (Fig. 4a). Ten coexpression modules were identified by WGCNA (Fig. 4b), among which the turquoise module was positively correlated with the contents of pelargonidin-3-O-glucoside (r = 0.67, *p* value = 3e-06), cyanidin-3-O-glucoside (r = 0.80, *p* value = 8e-11), cyanidin-3-O-rutinoside (r = 0.75, *p* value = 4e-08), and quercetin-3-O-rutinose (r = 0.83, *p* value = 6e-11). In addition, the turquoise module was positively correlated with the storage temperature of 10 °C, and the correlation coefficient was 0.90 (*p* value = 6e-15) (Fig. 4c). According to GO and KEGG enrichment of the candidate genes in the turquoise module (1416 genes in total), 33 genes were mapped to the flavonoid metabolism pathway, and 31 genes were mapped to the starch and sugar metabolism pathway (see Additional file 1: Figs. S1 and S2).

### Identification of candidate genes involved in anthocyanin biosynthesis

**Fig. 5:**
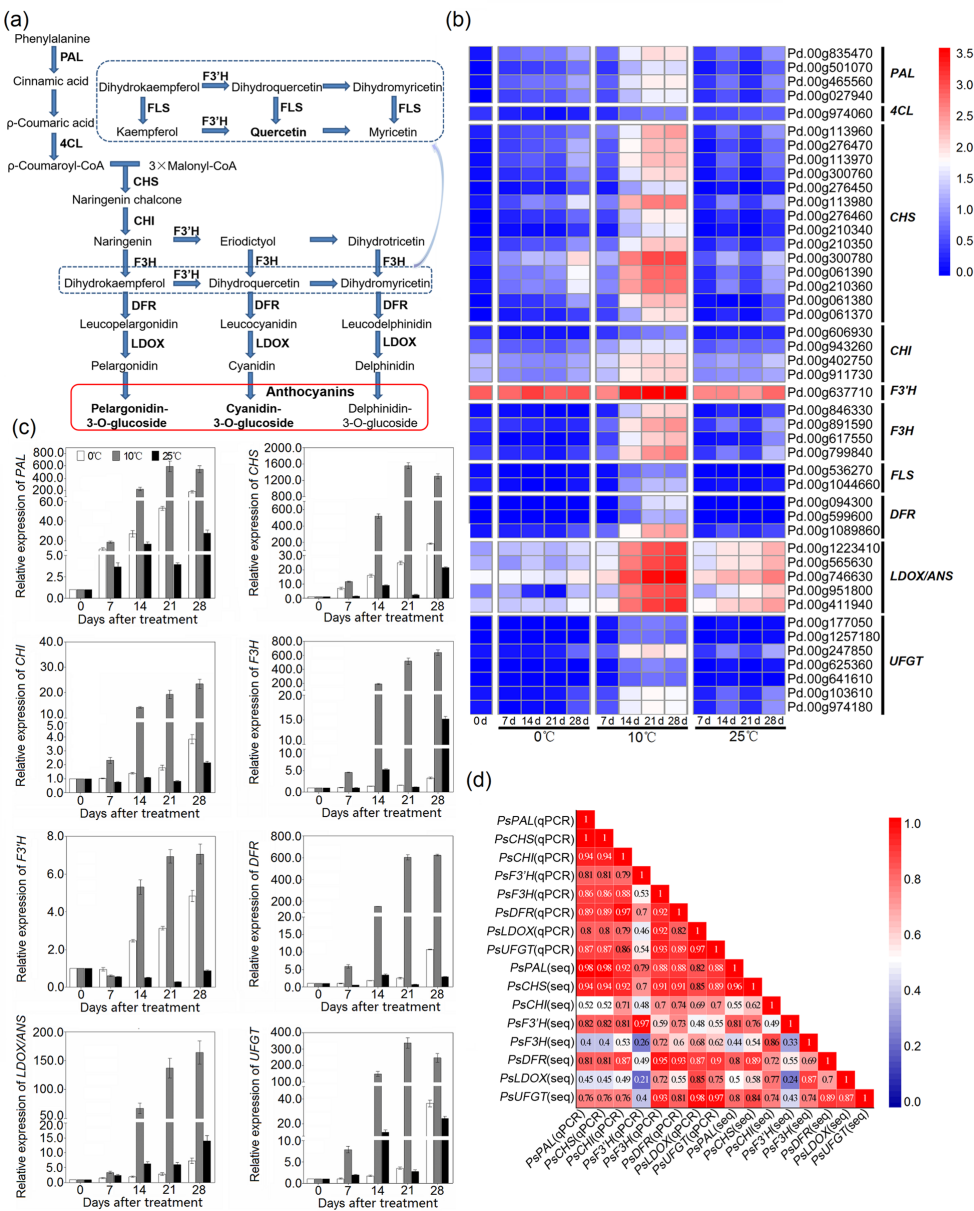
Analysis of genes related to anthocyanin biosynthesis in the turquoise module. **a** Anthocyanin biosynthetic pathway. The bold font indicates the genes obtained in the turquoise module and the anthocyanin components detected in the flesh of ‘Friar’ plum fruit. **b** Heatmap of the expression levels of differentially expressed genes (DEGs) involved in anthocyanin biosynthesis. **c** qRT–PCR detection of anthocyanin synthesis-related structural genes, *PsPAL* (Pd.00 g835470), *PsCHS* (Pd.00 g300780), *PsCHI* (Pd.00 g402750), *PsF3H* (Pd.00 g891590), *PsF3’H* (Pd.00 g637710), *PsDFR* (Pd.00 g1089860), *PsLDOX* (Pd.00 g746630), and *PsUFGT* (Pd.00 g247850). **d** Correlation analysis of the expression profiles in qRT–PCR (qPCR) and transcriptome data (RNA-seq) (N = 13, |r| > 0.55 represents a significant correlation between the two sets of data)

A total of 43 structural genes involved in anthocyanin biosynthesis were obtained in the turquoise module, and a heatmap of their expression profiles in the flesh of ‘Friar’ plum fruit was drawn based on their FPKM value (log10(FPKM+1)) (Fig. 5). The 43 structural genes from all major steps of the anthocyanin biosynthesis pathway were distributed as follows: four phenylalanine ammonia-lyase genes (*PAL*), one 4-coumarate: coenzyme A ligase (*4CL*), fourteen chalcone synthase genes (*CHS*), four chalcone isomerase genes (*CHI*), one flavonoid-3′-hydroxylase gene (*F3’H*), four flavanone 3-hydroxylase genes (*F3H*), three dihydroflavonol 4-reductase genes (*DFR*), five leucoanthocyanidin dioxygenase/anthocyanin synthase genes (*LDOX/ANS*) and seven UDP-glucose: flavonoid 3-O-glucosyltransferase genes (*UFGT*).

Furthermore, the expression patterns of eight representative structural genes involved in anthocyanin biosynthesis, *PsPAL* (Pd.00 g835470), *PsCHS* (Pd.00 g300780), *PsCHI* (Pd.00 g402750), *PsF3H* (Pd.00 g891590), *PsF3’H* (Pd.00 g637710), *PsDFR* (Pd.00 g1089860), *PsLDOX* (Pd.00 g746630) and *PsUFGT* (Pd.00 g247850), were studied via qRT–PCR, and the transcripts of these genes were significantly higher in the flesh of plum fruit stored at 10 °C than in that stored at 0 °C and 25 °C (Fig. 5c), which was consistent with the results of transcriptome analysis based on the correlation analysis (Fig. 5d).

### Identification of genes involved in carbohydrate metabolism

**Fig. 6:**
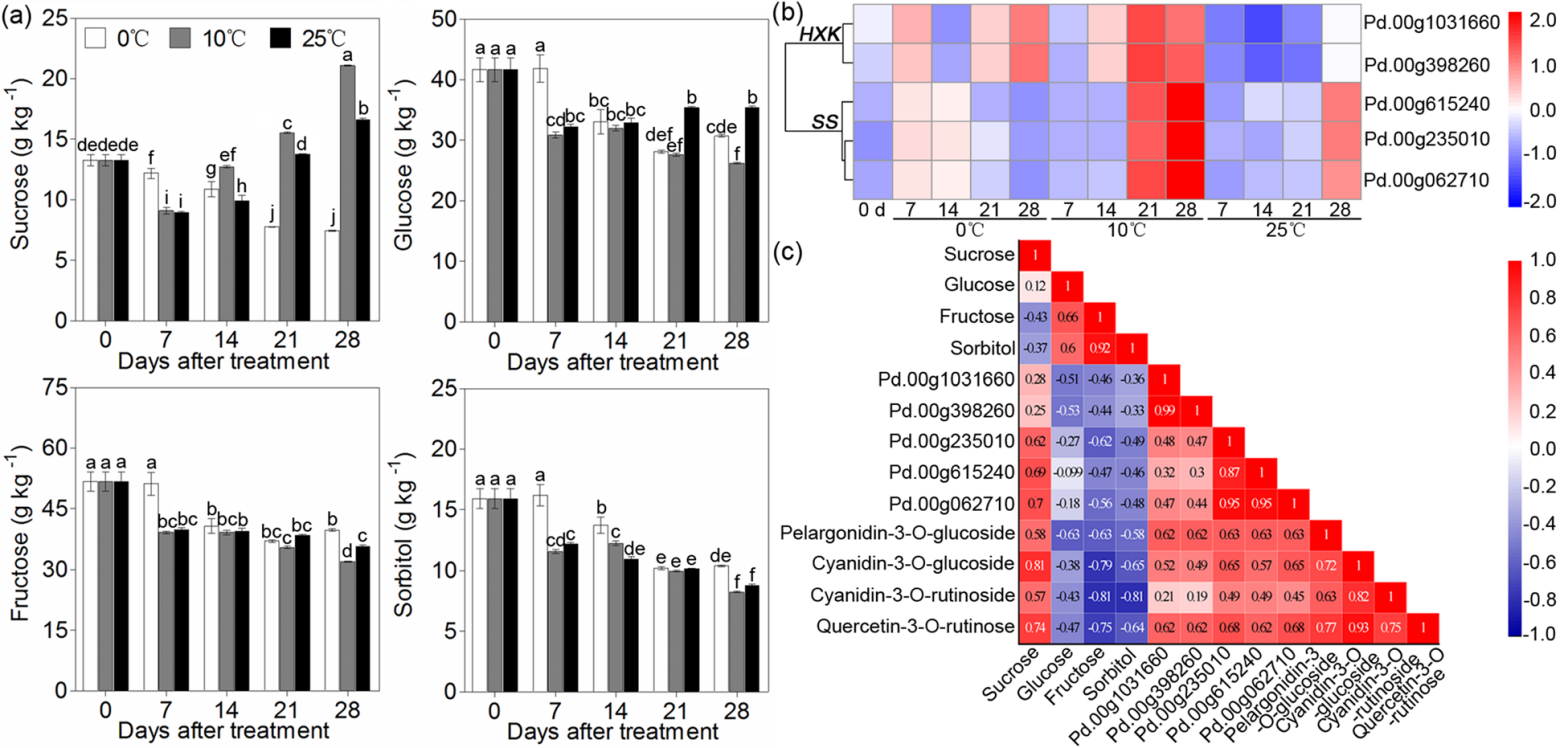
Soluble sugar content and expression pattern of carbohydrate metabolism-related genes under different storage temperatures. **a** Glucose, fructose, sorbitol and sucrose contents in ‘Friar’ plum fruit under different storage temperatures. **b** Heatmap of the expression levels of carbohydrate metabolism-related genes in the turquoise module. **c** Correlation analysis of anthocyanin content, soluble sugar content and expression profiles of carbohydrate metabolism-related genes (N = 13, |r| > 0.55 represents a significant correlation between the two sets of data). Different lowercase letters above the bars indicate significant differences (*P* < 0.05), which were obtained based on one-way ANOVA by LSD and DUNCAN tests

Carbohydrates are considered substrates for anthocyanin synthesis, and the change in soluble sugar content was detected by HPLC. The contents of glucose, fructose and sorbitol showed downward trends at three different storage temperatures. The sucrose content decreased in the flesh of plum fruit during 0 °C storage but increased during 10 °C and 25 °C storage; moreover, it was higher at 0 °C than at 25 °C (Fig. 6a). In the turquoise module, which was related to anthocyanin synthesis, five genes were involved in starch and sugar metabolism, including two hexokinases (HXKs) and three sucrose synthases (SSs) (Fig. 6b). Correlation analysis showed that the expression patterns of the *SS* genes were positively correlated with sucrose content and anthocyanin content (Fig. 6c), suggesting that higher expression levels of these genes were beneficial to carbohydrate metabolism, which contributed to anthocyanin accumulation in the flesh under storage at 10 °C.

### Screening potential transcription factors that regulate anthocyanin synthesis

**Fig. 7:**
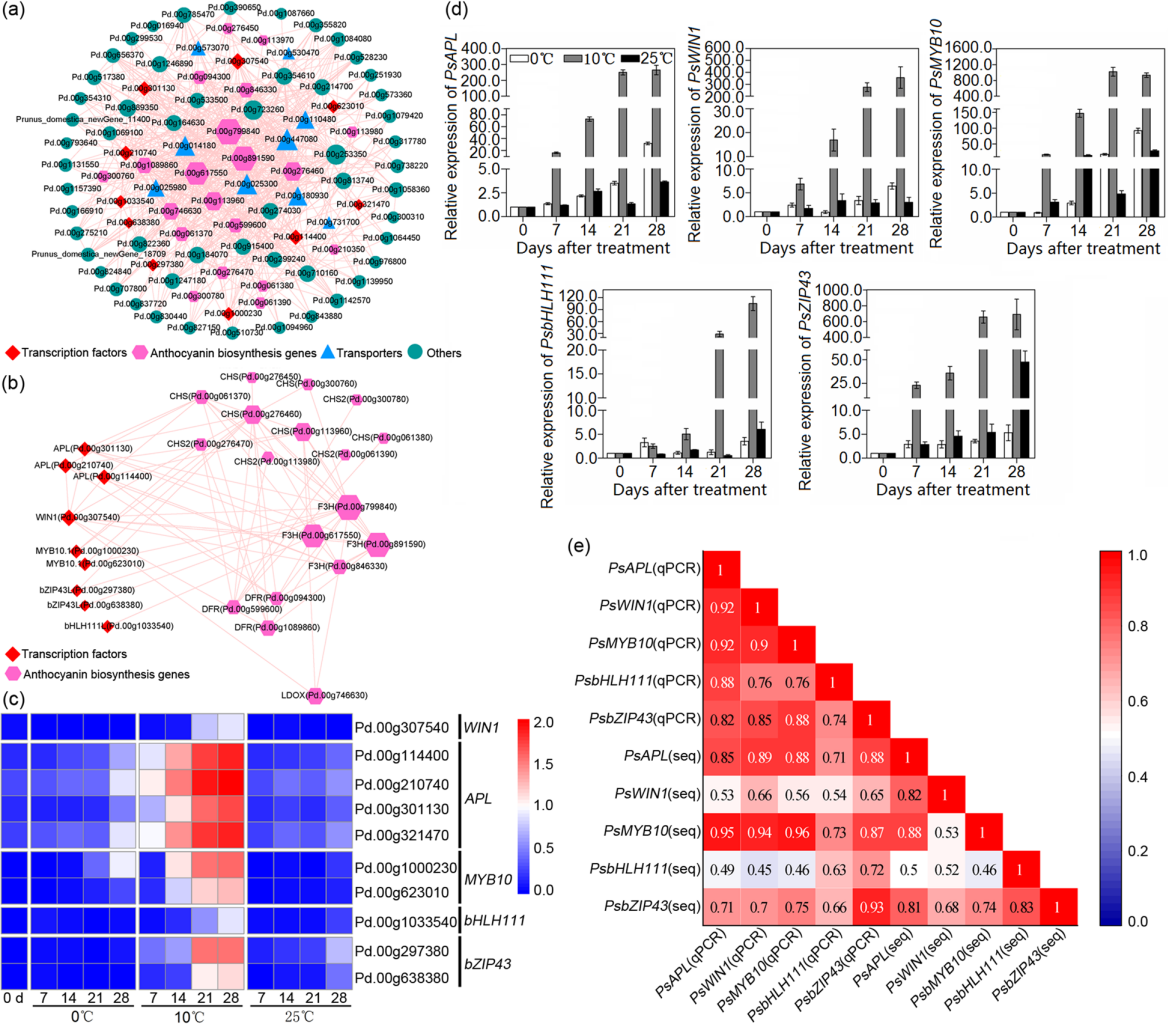
Coexpression network analysis of the potential key genes in turquoise modules based on WGCNA. **a** Coexpression network of the genes (weight > 0.5). **b** Coexpression network of transcription factors and anthocyanin biosynthesis-related structural genes. **c** Heatmap of the expression levels of the candidate transcription factors. **d** qRT–PCR detection of candidate transcription factor genes *PsAPL* (Pd.00 g301130), *PsWIN1* (Pd.00 g307540), *PsMYB10* (Pd.00 g623010), *PsbHLH111* (Pd.00 g1033540), and *PsbZIP43* (Pd.00 g297380). **e** Correlation analysis of the expression profiles in qRT–PCR (qPCR) and transcriptome data (RNA-seq) (N = 13, |r| > 0.55 represents a significant correlation between the two sets of data)

To further explore the molecular regulatory mechanism of anthocyanin biosynthesis in the flesh of ‘Friar’ plum fruit, a coexpression network was constructed based on the genes present in the turquoise module. In the network, three *F3H* genes (Pd.00 g799840, Pd.00 g891590 and Pd.00 g617550) and two *CHS* genes (Pd.00 g276460 and Pd.00 g113960) were identified as hub genes (Fig. 7a), and five transcription factor genes, *MYB10*, *APL*, *WIN1*, *bHLH111* and *bZIP43,* were found to be coexpressed with anthocyanin biosynthesis-related genes. Among the five transcription factor genes, *MYB10* and *WIN1* were closely related to *CHS*, *F3H*, *DFR* and *LDOX*, *APL* was closely related to *CHS*, *F3H* and *DFR*, *bHLH111* was only closely related to *F3H*, and *bZIP43* was closely related to *CHS* and *F3H*. In addition, there was a positive correlation between *APL* and *WIN1* (Fig. 7b).

The heatmap based on the transcriptome data showed that the expression levels of *APL*, *WIN1*, *MYB10*, *bHLH111* and *bZIP43* were obviously upregulated in the fruit stored at 10 °C, while there was little change in the fruit stored at 0 °C and 25 °C (Fig. 7c). Meanwhile, the qRT–PCR results indicated that the transcripts of these five detected genes in the fruit stored at 10 °C were markedly higher than those in the fruit stored at 0 °C and 25 °C (Fig. 7d), which was consistent with the transcriptome analysis results based on the correlation analysis (Fig. 7e). Furthermore, the expression patterns of the five transcription factor genes were positively correlated with the changes in the expression levels of the structural genes and the anthocyanin content (see Additional file 1: Fig. S3). It was proposed that *APL*, *WIN1*, *MYB10*, *bHLH111* and *bZIP43* might be involved in anthocyanin accumulation by regulating the expression of structural genes associated with anthocyanin biosynthesis.

## Discussion

### Intermediate temperature is beneficial to promote anthocyanin accumulation in plum fruit

Temperature is an important environmental factor that affects anthocyanin accumulation. Low temperature (4 °C) can significantly induce anthocyanin accumulation in *Arabidopsis* seedlings in the presence of light [24]. Intermediate temperature (16 °C) effectively leads to reddening in the leaves of apple and begonia [29, 30], and 15 °C treatment promotes anthocyanin accumulation in grape peel [31]. For postharvest fruits, intermediate temperature also promotes the process of anthocyanin accumulation in peach, kiwifruit, sweet orange and plum fruits [26–28, 32–35]. In this work, the flesh significantly accumulated anthocyanins and appeared red on the 14th day of storage when the fruit was stored at 10 °C, while the flesh of the fruit stored at 0 °C and 25 °C showed lower anthocyanin contents and did not redden within 28 days of storage (Fig. 1a). This was similar to the findings of a previous report [27], in which the colour of flesh rapidly changed to red once the ‘Friar’ plum fruit was stored at 5 °C and 15 °C but reddened slowly at 0 °C or did not turn red at 25 °C. It also suggested that intermediate temperature was prone to accumulate anthocyanin in the flesh of ‘Friar’ plum fruit.

Anthocyanidins are widely distributed among diverse plant species. These are flavonoids that are usually present in the form of six common anthocyanidins, pelargonidin, cyanidin, delphinidin, peonidin, petunidin and malvidin. Anthocyanidins are very unstable; they can integrate with monosaccharides and disaccharides by glycosylation and then form various stable water-soluble anthocyanins [36, 37]. In this work, four anthocyanin components were identified in the flesh of ‘Friar’ plum fruit, and moreover, their contents significantly increased as the flesh reddened in plum fruit (Fig. 1 a and Fig. 2 a-d). This result was consistent with previous findings in plum fruit [27], indicating that flesh reddening under 10 °C storage was the result of the accumulation of these four anthocyanin components. The upregulation of structural genes resulted in anthocyanin accumulation [23, 24, 34, 38]. To understand the regulation of anthocyanin biosynthesis in ‘Friar’ plum fruit under intermediate temperature storage, 43 differentially expressed structural genes were found based on the transcriptome data. All of the candidate genes (*PAL*, *4CL*, *CHS*, *CHI*, *F3’H*, *F3H*, *FLS*, *DFR*, *LDOX/ANS* and *UFGT*) were completely mapped to the anthocyanin biosynthesis pathway and covered almost all of the key genes of this pathway (Fig. 5a). Moreover, the flesh of fruit stored at 10 °C showed rapid reddening and demonstrated higher expression levels of structural genes, which were positively correlated with the accumulation of quercetin and anthocyanin (Fig. 2 and Fig. 5 c and Fig. S3). Therefore, it was confirmed that intermediate temperature (10 °C) storage could promote the red colour development of flesh by increasing the transcripts of anthocyanin synthesis-related structural genes. Carbohydrates are an important substrate for anthocyanidin transformation into anthocyanins, and they have been considered a signal for regulating anthocyanin biosynthesis [39]. Galactose is the main substrate for anthocyanin synthesis in apple, and glucose can activate the activity of hexokinase (MdHXK1), which subsequently phosphorylates the MdbHLH3 transcription factor to regulate fruit reddening [40, 41]. In grapes, carbohydrates can induce the expression of *F3H* and then stimulate anthocyanin accumulation [42]. Among the detected carbohydrates in this study, only the sucrose content significantly increased under 10 °C storage once the flesh appeared to redden at Day 14 (Fig. 1a and Fig. 6); in addition, the sucrose synthase (SS) genes showed higher expression levels. Thus, sucrose might be involved in anthocyanin synthesis as a substrate or signal in ‘Friar’ plum fruit stored at 10 °C.

### Transcription factors involved in anthocyanin accumulation in plum fruit

Anthocyanin biosynthesis can be regulated by various transcription factors, and the MBW complex, which regulates anthocyanin accumulation by triggering structural genes, is well studied [43–46]. MYB10 has been proven to be involved in anthocyanin synthesis by regulating *DFR*, *LDOX* and *UFGT* in apple, pear, nectarine, apricot, sweet cherry, strawberry and mangosteen [22, 47–51], and *PsMYB10.1* can participate in anthocyanin accumulation by regulating *PsANS*, *PsUFGT* and *PsGST* in the pericarp of the postharvest ‘Akihime’ plum *(Prunus salicina* Lindl.) under 20 °C/light treatment [32]*.* In addition, transient overexpression of *PaMYB10* increased anthocyanin content in the peel of apricot fruit [52]. Heterologous overexpression of *PpMYB10.1*/*PpbHLH3* and *PpMYB10.3*/*PpbHLH3* activated anthocyanin production in tobacco leaves by upregulating *NtCHS*, *NtDFR* and *NtUFGT* [18, 53]. Two bHLH transcription factors, MdbHLH3 and MdbHLH33, have been confirmed to regulate *DFR* to promote anthocyanin biosynthesis by interacting with MYB10 in apple [54], and MdbHLH3 could regulate low temperature-induced anthocyanin synthesis by binding to the LTR (low temperature response) element of the promotor of *MdMYBPA1* [55]. Moreover, bHLH3 could directly regulate the expression of structural genes to facilitate anthocyanin accumulation [23, 49]. In the present study, both transcriptome analysis and qPCR quantitative detection indicated that the expression level of *MYB10* significantly increased once anthocyanin accumulated in the flesh of ‘Friar’ plum fruit under 10 °C storage, and this was coordinated with the expression patterns of structural genes (Fig. 7). Thus, the transcription factor MYB10 was involved in regulating anthocyanin accumulation in ‘Friar’ plum fruit under intermediate temperature storage. Except for *MYB10*, the expression profiles of *APL* and *bHLH111* were also found to be closely related to anthocyanin accumulation and changes in the structural genes, and moreover, the expression patterns of *APL* and *bHLH111* were similar to *MYB10*, indicating that the transcription factors *APL* and *bHLH111* were also involved in regulating anthocyanin synthesis in ‘Friar’ plum fruit. The proposed function of APL is that it participates in the response to phosphorus and nitrogen deficiency and promotes flowering in *Arabidopsis thaliana* [56, 57]. bHLH111 has been reported to determine the competence of the pericycle for lateral root initiation in *Arabidopsis thaliana* [58]. However, their roles in regulating anthocyanin accumulation in postharvest fruit are unclear and need to be studied further.

The ethylene-responsive transcription factor WIN1 has been reported to be involved in wax biosynthesis and defence responses in *Arabidopsis thaliana* [59, 60]. In this study, 10 °C storage induced higher expression of *WIN1*, which was positively correlated with anthocyanin accumulation and the expression patterns of structural genes (Fig. 7); thus, *WIN1* participated in the regulation of anthocyanin biosynthesis. Previous studies have shown that MdEIL1 (EIN3-LIKE1) and MdERF1b (ETHYLENE RESPONSE FACTOR1b) promote anthocyanin synthesis by regulating transcription factors such as MdMYB1, MdMYB9 and MdMYB11 in apple [61, 62]. It was found that *WIN1* was closely related to *APL* based on the analysis of the coexpression network in the present study, so it was hypothesized that WIN1 might regulate anthocyanin synthesis by interacting with APL. The transcription factor bZIP43 was found to be associated with somatic embryogenesis induction and involved in stress responses by interacting with bHLH109 in *Arabidopsis* [63]. Considering that anthocyanin accumulation is one of the responses to environmental stress in plants, the transcription factor bZIP43 probably plays a role in regulating anthocyanin synthesis in ‘Friar’ plum fruit under intermediate storage temperatures.

## Conclusions

This study found that an intermediate storage temperature (10 °C) accelerated the process of anthocyanin accumulation in ‘Friar’ plum fruit. Cyanidin-3-O-glucoside and cyanidin-3-O-rutinoside contributed to the red colour of flesh. Based on the transcriptome and metabolite profile and subsequent WGCNA, 43 anthocyanin biosynthesis genes and two carbohydrate metabolism-associated genes were identified. According to the gene coexpression network constructed by WGCNA, *F3H* and *CHS* were recognized as hub genes, and five transcription factors, *APL*, *WIN1*, *MYB10*, *bZIP43* and *bHLH111,* were proposed to be involved in anthocyanin biosynthesis by regulating the structural genes.

## Methods

### Materials and treatments

‘Friar’ plum fruit was harvested at commercial maturity from Yi County, Baoding City, Hebei Province. The plum fruit (average single fruit weight 110 g ± 9.25 g; soluble solids content 10.4% ± 0.96% and firmness 77.94 N ± 6.44 N) without visible defects were divided into three groups and each group (approximately 450 plums) were averagely placed in three cardboard boxes (44.5 × 31.5 × 20.5 cm^3^), and then stored in the dark at 25 ± 1 °C (25 °C), 10 ± 0.5 °C (10 °C), and 0 ± 0.5 °C (0 °C) with 85–90% relative humidity. The quality assays were performed at 7 d intervals during storage, with three repetitions of each treatment with 5 fruits per repetition, and the flesh samples were quickly frozen in liquid nitrogen and stored at −80 °C until use.

### Determination of fruit quality

Firmness at two equidistant points on the equatorial region of fruit with skin removal was determined using a GY-4 digital fruit hardness metre (TOP Instruments Co., Hangzhou, Zhejiang, China), and firmness was calculated and expressed in Newtons (N). The soluble solids content (SSC) was measured by a PAL-1 pocket digital refractometer (ATAGO CO., LTD., Tokyo, Japan).

### Determination of soluble sugar content

Five grams of flesh powder ground in liquid nitrogen was weighed, and then 5 mL of 85% ethanol solution was added for ultrasonic extraction at 40 °C for 1 h, followed by centrifugation at 8000 g for 10 min. The supernatant was collected. The extraction was repeated once more, and the supernatant was combined. After filtration through a 0.45 μm PTFE microporous membrane, the combined supernatant was collected for sugar analysis by HPLC (LC-20AT, SHIMADZU CORP., Kyoto, Japan). The measurement conditions were as follows: chromatographic column: Bio–Rad Aminex HPX-87H, detector: differential RID, mobile phase: 5 mM sulfuric acid, flow rate: 0.3 mL/min, column temperature: 40 °C, injection volume: 10 μL.

### Determination of phenolic acid and flavonoid contents

#### Phenolic acid extraction

Phenolic acid extracts were carried out according to Wang et al. [64] with minor modifications. Briefly, 2 g of freeze-dried powder was extracted with 10 mL of 80% methanol containing 0.5% hydrochloric acid. Afterwards, the mixture was ultrasonicated for 30 min and then centrifuged at 10000 × g at 4 °C for 10 min. Finally, the supernatant was collected. The extraction was repeated twice, and the combined supernatants were evaporated until almost dry at 50 °C under a gentle nitrogen stream. The resultant dry residues were redissolved in 5 mL of 50% (v/v) methanol/ultrapure water and filtered through a 0.22 μm PTFE membrane filter (Pall, MI, USA).

#### Flavonoid extraction

Flavonoid extracts were performed according to the method of Gao et al. [65] with some modifications. Two grams of the freeze-dried powder of plum flesh was extracted with 30 mL of 80% methanol in the dark for 24 h at −20 °C. Afterwards, the resultant mixture was centrifuged at 10000 × g at 4 °C for 10 min, and then the supernatant was immediately collected for analysis. The flavonoid contents were determined using UPLC–MS/MS after filtration through 0.22 μm PTFE membrane filters (Pall, MI, USA).

#### UPLC–MS/MS analysis

Phenolic and flavonoid analyses were performed as described previously by Gao et al*.* [65]. The samples were analysed by using an Acquity UPLC system (Waters, Milford, MA) with a triple quadrupole mass spectrometer (TQ-S, Waters Micromass, Manchester, UK). An acquity HSS C18 column (1.8 μm particle size; 2.1 × 150 mm; Waters, Milford, MA, USA) was used to perform chromatographic separation. The column and sample managers were maintained at 40 °C and 10 °C, respectively. The mobile phase used for the separation consisted of (A) 0.1% (v/v) formic acid in water and (B) 0.1% (v/v) formic acid in acetonitrile. Samples were eluted according to a linear gradient: 0.5–4.5 min, 5–30% B; 4.5–9.0 min, 30–90%; 9.0–10.0 min, 0.5% B, and a flow rate of 0.3 mL min^−1^.

The mass spectrometer was operated in both positive and negative ionization modes, depending on the structure and properties of the compounds. The parameters were as follows: capillary voltage, + 2.5 kV/−1.0 kV; source temperature, 150 °C; desolvation temperature, 500 °C; cone gas flow, 150 L h^−1^; and desolvation gas flow, 1000 L h^−1^. Detection was carried out in multiple reaction monitoring (MRM) mode. All analyte-dependent parameters were conducted according to our previously published study [65]. MassLynxTM 4.1 software (Waters) was used for data acquisition and processing. Quantitative determinations were performed using the standard curves generated from individual compounds in serial dilutions (1–500 ng mL^−1^).

#### Transcriptome sequencing and WGCNA

Total RNA was extracted from ‘Friar’ flesh samples stored at 0 °C, 10 °C and 25 °C for 7 days, 14 days, 21 days and 28 days (three biological repetitions), and RNA concentration and purity were measured using a NanoDrop 2000 (Thermo Fisher Scientific, Wilmington, DE). RNA integrity was assessed using the RNA Nano 6000 Assay Kit of the Agilent Bioanalyzer 2100 system (Agilent Technologies, CA, USA). A total amount of 1 μg of RNA per sample was used as input material for the RNA sample preparations. Sequencing libraries were generated using the NEBNext UltraTM RNA Library Prep Kit for Illumina (NEB, USA) following the manufacturer’s recommendations, and index codes were added to attribute sequences to each sample. Library quality was assessed with the 2100 Bioanalyzer system. After the library inspection was qualified, different libraries were pooled according to the target data volume and sequenced by the Illumina platform. The off-board data were filtered to obtain clean data, compared with the reference genome of European plum [66] to obtain mapped data, and evaluated for library quality, such as insert length tests and randomness tests. Then, structural-level analysis, such as alternative splicing analysis, new gene discovery and gene structure optimization, was carried out. The expression levels of differentially expressed genes, functional annotation and functional enrichment of differentially expressed genes were analysed. Finally, the transcriptome data and phenotypic data were analysed by WGCNA using R language.

#### RNA extraction and qPCR analysis

Total RNA from flesh was extracted by the CTAB method [67]. RNA (0.8 μg, OD260:OD280 between 1.80 and 2.0, OD260:OD230 > 1.5, no obvious degradation by electrophoresis) was used for reverse transcription by PrimeScript™ RT Reagent Kit with gDNA Eraser (Takara Biomedicals, Dalian, China). The products were diluted 15 times with nuclease-free water and then subjected to real-time fluorescence quantitative PCR (qPCR) with a TB Green® Premix Ex Taq™ II (Tli RNaseH Plus) kit (TaKaRa Biomedicals).

Quantitative real-time (qRT)-PCR assays were conducted using an Applied Biosystems 7500 Fast Real-Time PCR System. The reaction system was 20 μL, including 10 μL of 2X Green Premix Ex Taq II (Tli RNaseH Plus), 0.8 μL each of gene specific upstream primer and downstream primer, 0.4 μL of ROX Reference Dye II (50X), 2 μL of diluted cDNA, and 6 μL of nuclease-free water. The running program was set as follows: 30 s at 95 °C for one cycle, 5 s at 95 °C and 34 s at 60 °C for 40 cycles. *PsACTIN7* was used as the internal reference. The primers used in this paper are listed in Additional file 1: Table S1. The relative expression levels of genes were calculated according to the 2^-△△Ct^ method.

### Statistical analysis

Each experiment was performed in three replicates. Experimental results were analysed using GraphPad Prism 8, Origin 2021, IBM SPSS Statistics 23, RStudio, and Cytoscape 3.7.1 software. Error bars denote standard deviations. Different lowercase letters above the bars indicate significant differences (*P* < 0.05), which were obtained based on one-way ANOVA by LSD and DUNCAN tests using IBM SPSS Statistics 23 software.

## Supplementary Information

**Additional file 1: Figure S1**. GO enrichment of the genes in turquoise module. **Figure S2.** KEGG enrichment of the genes in turquoise module. **Figure S3.** Correlation analysis of expression profiles of anthocyanin biosynthesis related genes, anthocyanin components and quercetin content. **Table S1.** The primers used in this study.

## Data Availability

The datasets supporting the conclusions of this article are included within the article (and its additional file(s).

## Abbreviations

WGCNA: Weighted gene co-expression correlation network analysis
ROS: Reactive oxygen species
EBGs: Early biosynthesis genes
LBGs: Late biosynthesis genes
PAL: Phenylalnine ammonialyase
CHS: Chalcone synthase
CHI: Chalcone isomerase
F3H: Flavanone 3-hydroxylase
F3’H: Flavonoid-3′-hydroxylase
DFR: Dihydroflavonol 4-reductase
LDOX/ANS: Leucoanthocyanidin dioxygenase/Anthocyanin synthetase
UFGT: UDP-glucose: flavonoid 3-O-glucosyltransferase
MBW: MYB-bHLH-WD40
COP1: CONSTITUTIVE PHOTOMORPHOGENIC 1
JAZ: JASMONATE ZIM-DOMAIN
NAC: NAM, ATAF1/2, CUC2
SPL: SQUAMOSA promoter-binding protein-like
HXK: Hexokinase
SS: sucrose synthases
APL: MYB family transcription factor APL
MYB10: MYB10 transcription factor
WIN1: Ethylene-responsive transcription factor WIN1
bZIP43: Basic leucine zipper 43-like
bHLH111: Transcription factor bHLH111-like isoform X2
LTR: Low temperature response
MdEIL1: EIN3-LIKE1
MdERF1b: ETHYLENE RESPONSE FACTOR1b
DAT: Days after treatment
SSC: Soluble solids content
FPKM: Fragments per kilobase of exon model per million mapped fragments
DEGs: Differentially expressed genes
UPLC–MS/MS: ultra-performance liquid chromatography–tandem mass spectrometry
HPLC: High performance liquid chromatography

## References

[1] Bai S, Tao R, Yin L, Ni J, Yang Q, Yan X (2019). Two B-box proteins, PpBBX18 and PpBBX21, antagonistically regulate anthocyanin biosynthesis via competitive association with Pyrus pyrifolia ELONGATED HYPOCOTYL 5 in the peel of pear fruit. Plant J.

[2] Davies KM, Albert NW, Schwinn KE (2012). From landing lights to mimicry: the molecular regulation of flower colouration and mechanisms for pigmentation patterning. Funct Plant Biol.

[3] Lev-Yadun S, Gould KS, Winefield C, Davies K, Gould K (2008). Role of anthocyanins in plant defence. Anthocyanins.

[4] Tsuda T (2012). Dietary anthocyanin-rich plants: biochemical basis and recent progress in health benefits studies. Mol Nutr Food Res.

[5] Pelletier MK, Murrell JR, Shirley BW (1997). Characterization of flavonol synthase and leucoanthocyanidin dioxygenase genes in *Arabidopsis*. Further evidence for differential regulation of "early" and "late" genes. Plant Physiol.

[6] Jeong SW, Das PK, Jeoung SC, Song JY, Lee HK, Kim YK (2010). Ethylene suppression of sugar-induced anthocyanin pigmentation in *Arabidopsis*. Plant Physiol.

[7] Ni J, Zhao Y, Tao R, Yin L, Gao L, Strid A (2020). Ethylene mediates the branching of the jasmonate-induced flavonoid biosynthesis pathway by suppressing anthocyanin biosynthesis in red Chinese pear fruits. Plant Biotechnol J.

[8] Saito K, Yonekura-Sakakibara K, Nakabayashi R, Higashi Y, Yamazaki M, Tohge T (2013). The flavonoid biosynthetic pathway in *Arabidopsis*: structural and genetic diversity. Plant Physiol Biochem.

[9] Gonzalez A, Zhao M, Leavitt JM, Lloyd AM (2008). Regulation of the anthocyanin biosynthetic pathway by the TTG1/bHLH/Myb transcriptional complex in *Arabidopsis* seedlings. Plant J.

[10] Xu W, Dubos C, Lepiniec L (2015). Transcriptional control of flavonoid biosynthesis by MYB-bHLH-WDR complexes. Trends Plant Sci.

[11] Zimmermann IM, Heim MA, Weisshaar B, Uhrig JF (2004). Comprehensive identification of *Arabidopsis thaliana* MYB transcription factors interacting with R/B-like BHLH proteins. Plant J.

[12] Koes R, Verweij W, Quattrocchio F (2005). Flavonoids: a colorful model for the regulation and evolution of biochemical pathways. Trends Plant Sci.

[13] Gonzalez A, Brown M, Hatlestad G, Akhavan N, Smith T, Hembd A (2016). TTG2 controls the developmental regulation of seed coat tannins in *Arabidopsis* by regulating vacuolar transport steps in the proanthocyanidin pathway. Dev Biol.

[14] Gou JY, Felippes FF, Liu CJ, Weigel D, Wang JW (2011). Negative regulation of anthocyanin biosynthesis in *Arabidopsis* by a miR156-targeted SPL transcription factor. Plant Cell.

[15] Maier A, Schrader A, Kokkelink L, Falke C, Welter B, Iniesto E (2013). Light and the E3 ubiquitin ligase COP1/SPA control the protein stability of the MYB transcription factors PAP1 and PAP2 involved in anthocyanin accumulation in *Arabidopsis*. Plant J.

[16] Qi T, Song S, Ren Q, Wu D, Huang H, Chen Y (2011). The Jasmonate-ZIM-domain proteins interact with the WD-repeat/bHLH/MYB complexes to regulate Jasmonate-mediated anthocyanin accumulation and trichome initiation in *Arabidopsis thaliana*. Plant Cell.

[17] Verweij W, Spelt CE, Bliek M, de Vries M, Wit N, Faraco M (2016). Functionally similar WRKY proteins regulate vacuolar acidification in petunia and hair development in *Arabidopsis*. Plant Cell.

[18] Zhou H, Lin-Wang K, Wang H, Gu C, Dare AP, Espley RV (2015). Molecular genetics of blood-fleshed peach reveals activation of anthocyanin biosynthesis by NAC transcription factors. Plant J.

[19] Bai S, Saito T, Moriguchi T, Honda C, Hatsuyama Y, Ito A (2014). An apple B-box protein, MdCOL11, is involved in UV-B- and temperature-induced anthocyanin biosynthesis. Planta..

[20] Crifo T, Puglisi I, Petrone G, Recupero GR, Lo Piero AR (2011). Expression analysis in response to low temperature stress in blood oranges: implication of the flavonoid biosynthetic pathway. Gene..

[21] Das PK, Shin DH, Choi SB, Park YI (2012). Sugar-hormone cross-talk in anthocyanin biosynthesis. Mol Cell.

[22] Feng S, Wang Y, Yang S, Xu Y, Chen X (2010). Anthocyanin biosynthesis in pears is regulated by a R2R3-MYB transcription factor PyMYB10. Planta..

[23] Xie XB, Li S, Zhang RF, Zhao J, Chen YC, Zhao Q (2012). The bHLH transcription factor MdbHLH3 promotes anthocyanin accumulation and fruit colouration in response to low temperature in apples. Plant Cell Environ.

[24] Zhang Y, Liu Z, Liu R, Hao H, Bi Y (2011). Gibberellins negatively regulate low temperature-induced anthocyanin accumulation in a HY5/HYH-dependent manner. Plant Signal Behav.

[25] Zhang C, Jia H, Wu W, Wang X, Fang J, Wang C (2015). Functional conservation analysis and expression modes of grape anthocyanin synthesis genes responsive to low temperature stress. Gene..

[26] Zhu YC, Zhang B, Allan AC, Lin-Wang K, Zhao Y, Wang K (2020). DNA demethylation is involved in the regulation of temperature-dependent anthocyanin accumulation in peach. Plant J.

[27] Wang R, Wang L, Yuan S, Li Q, Pan H, Cao J (2018). Compositional modifications of bioactive compounds and changes in the edible quality and antioxidant activity of 'Friar' plum fruit during flesh reddening at intermediate temperatures. Food Chem.

[28] Wang L, Sang W, Xu R, Cao J (2020). Alteration of flesh color and enhancement of bioactive substances via the stimulation of anthocyanin biosynthesis in 'Friar' plum fruit by low temperature and the removal. Food Chem.

[29] Song T, Li K, Wu T, Wang Y, Zhang X, Xu X (2019). Identification of new regulators through transcriptome analysis that regulate anthocyanin biosynthesis in apple leaves at low temperatures. PLoS One.

[30] Tian J, Han ZY, Zhang LR, Song TT, Zhang J, Li JY (2015). Induction of anthocyanin accumulation in crabapple (*Malus cv.*) leaves by low temperatures. HortScience..

[31] Azuma A, Yakushiji H, Koshita Y, Kobayashi S (2012). Flavonoid biosynthesis-related genes in grape skin are differentially regulated by temperature and light conditions. Planta..

[32] Fang Z, Lin-Wang K, Jiang C, Zhou D, Lin Y, Pan S (2021). Postharvest temperature and light treatments induce anthocyanin accumulation in peel of ‘Akihime’ plum (*Prunus salicina Lindl.*) via transcription factor PsMYB10.1. Postharvest Biol Technol.

[33] Li B, Xia Y, Wang Y, Qin G, Tian S (2017). Characterization of genes encoding key enzymes involved in anthocyanin metabolism of kiwifruit during storage period. Front Plant Sci.

[34] Sicilia A, Scialo E, Puglisi I, Lo Piero AR (2020). Anthocyanin biosynthesis and DNA methylation dynamics in sweet orange fruit [*Citrus sinensis L.* (*Osbeck*)] under cold stress. J Agric Food Chem.

[35] Yu M, Man Y, Wang Y (2019). Light- and temperature-induced expression of an R2R3-MYB gene regulates anthocyanin biosynthesis in red-fleshed kiwifruit. Int J Mol Sci.

[36] Tanaka Y, Sasaki N, Ohmiya A (2008). Biosynthesis of plant pigments: anthocyanins, betalains and carotenoids. Plant J.

[37] Alappat B, Alappat J (2020). Anthocyanin pigments: beyond aesthetics. Molecules..

[38] Ban Y, Honda C, Hatsuyama Y, Igarashi M, Bessho H, Moriguchi T (2007). Isolation and functional analysis of a MYB transcription factor gene that is a key regulator for the development of red coloration in apple skin. Plant Cell Physiol.

[39] Finkelstein RR, Gibson SI (2002). ABA and sugar interactions regulating development: cross-talk or voices in a crowd?. Curr Opin Plant Biol.

[40] Ban Y, Kondo S, Ubi BE, Honda C, Bessho H, Moriguchi T (2009). UDP-sugar biosynthetic pathway: contribution to cyanidin 3-galactoside biosynthesis in apple skin. Planta..

[41] Hu DG, Sun CH, Zhang QY, An JP, You CX, Hao YJ (2016). Glucose sensor MdHXK1 phosphorylates and stabilizes MdbHLH3 to promote anthocyanin biosynthesis in apple. PLoS Genet.

[42] Zheng Y, Tian L, Liu H, Pan Q, Zhan J, Huang W (2009). Sugars induce anthocyanin accumulation and flavanone 3-hydroxylase expression in grape berries. Plant Growth Regul.

[43] Baudry A, Heim MA, Dubreucq B, Caboche M, Weisshaar B, Lepiniec L (2004). TT2, TT8, and TTG1 synergistically specify the expression of *BANYULS* and proanthocyanidin biosynthesis in *Arabidopsis thaliana*. Plant J.

[44] Petroni K, Tonelli C (2011). Recent advances on the regulation of anthocyanin synthesis in reproductive organs. Plant Sci.

[45] Naing AH, Kim CK (2018). Roles of R2R3-MYB transcription factors in transcriptional regulation of anthocyanin biosynthesis in horticultural plants. Plant Mol Biol.

[46] He Q, Ren Y, Zhao W, Li R, Zhang L (2020). Low temperature promotes anthocyanin biosynthesis and related gene expression in the seedlings of purple head chinese cabbage (*Brassica rapa L.*). Genes (Basel).

[47] Palapol Y, Ketsa S, Lin-Wang K, Ferguson IB, Allan AC (2009). A MYB transcription factor regulates anthocyanin biosynthesis in mangosteen (*Garcinia mangostana L.*) fruit during ripening. Planta..

[48] Lin-Wang K, Bolitho K, Grafton K, Kortstee A, Karunairetnam S, McGhie TK (2010). An R2R3 MYB transcription factor associated with regulation of the anthocyanin biosynthetic pathway in Rosaceae. BMC Plant Biol.

[49] Ravaglia D, Espley RV, Henry-Kirk RA, Andreotti C, Ziosi V, Hellens RP (2013). Transcriptional regulation of flavonoid biosynthesis in nectarine (*Prunus persica*) by a set of R2R3 MYB transcription factors. BMC Plant Biol.

[50] Medina-Puche L, Cumplido-Laso G, Amil-Ruiz F, Hoffmann T, Ring L, Rodriguez-Franco A (2014). MYB10 plays a major role in the regulation of flavonoid/phenylpropanoid metabolism during ripening of *Fragaria x ananassa* fruits. J Exp Bot.

[51] Starkevic P, Paukstyte J, Kazanaviciute V, Denkovskiene E, Stanys V, Bendokas V (2015). Expression and anthocyanin biosynthesis-modulating potential of sweet cherry (*Prunus avium L.*) MYB10 and bHLH genes. PLoS One.

[52] Xi W, Feng J, Liu Y, Zhang S, Zhao G (2019). The R2R3-MYB transcription factor PaMYB10 is involved in anthocyanin biosynthesis in apricots and determines red blushed skin. BMC Plant Biol.

[53] Rahim MA, Busatto N, Trainotti L (2014). Regulation of anthocyanin biosynthesis in peach fruits. Planta..

[54] Espley RV, Hellens RP, Putterill J, Stevenson DE, Kutty-Amma S, Allan AC (2007). Red colouration in apple fruit is due to the activity of the MYB transcription factor, MdMYB10. Plant J.

[55] Wang N, Qu C, Jiang S, Chen Z, Xu H, Fang H (2018). The proanthocyanidin-specific transcription factor MdMYBPA1 initiates anthocyanin synthesis under low-temperature conditions in red-fleshed apples. Plant J.

[56] Todd CD, Zeng P, Huete AM, Hoyos ME, Polacco JC (2004). Transcripts of MYB-like genes respond to phosphorous and nitrogen deprivation in *Arabidopsis*. Planta..

[57] Abe M, Kaya H, Watanabe-Taneda A, Shibuta M, Yamaguchi A, Sakamoto T (2015). FE, a phloem-specific Myb-related protein, promotes flowering through transcriptional activation of FLOWERING LOCUS T and FLOWERING LOCUS T INTERACTING PROTEIN 1. Plant J.

[58] Zhang Y, Mitsuda N, Yoshizumi T, Horii Y, Oshima Y, Ohme-Takagi M (2021). Two types of bHLH transcription factor determine the competence of the pericycle for lateral root initiation. Nat Plants.

[59] Aharoni A, Dixit S, Jetter R, Thoenes E, van Arkel G, Pereira A (2004). The SHINE clade of AP2 domain transcription factors activates wax biosynthesis, alters cuticle properties, and confers drought tolerance when overexpressed in *Arabidopsis*. Plant Cell.

[60] Broun P, Poindexter P, Osborne E, Jiang CZ, Riechmann JL (2004). WIN1, a transcriptional activator of epidermal wax accumulation in *Arabidopsis*. PNAS..

[61] An JP, Wang XF, Li YY, Song LQ, Zhao LL, You CX (2018). EIN3-LIKE1, MYB1, and ETHYLENE RESPONSE FACTOR3 act in a regulatory loop that synergistically modulates ethylene biosynthesis and anthocyanin accumulation. Plant Physiol.

[62] Zhang J, Xu H, Wang N, Jiang S, Fang H, Zhang Z (2018). The ethylene response factor MdERF1B regulates anthocyanin and proanthocyanidin biosynthesis in apple. Plant Mol Biol.

[63] Nowak K, Gaj MD (2016). Stress-related function of bHLH109 in somatic embryo induction in *Arabidopsis*. J Plant Physiol.

[64] Wang M, Jiang N, Wang Y, Jiang D, Feng X (2017). Characterization of phenolic compounds from early and late ripening sweet cherries and their antioxidant and antifungal activities. J Agric Food Chem.

[65] Gao Y, Wang M, Jiang N, Wang Y, Feng X (2019). Use of ultra-performance liquid chromatography-tandem mass spectrometry on sweet cherries to determine phenolic compounds in peel and flesh. J Sci Food Agric.

[66] Zhebentyayeva T, Shankar V, Scorza R, Callahan A, Ravelonandro M, Castro S (2019). Genetic characterization of worldwide *Prunus domestica* (plum) germplasm using sequence-based genotyping. Hortic Res.

[67] Gasic K, Hernandez A, Korban SS (2004). RNA extraction from different apple tissues rich in polyphenols and polysaccharides for cDNA library construction. Plant Mol Biol Report.

