# Supplementary material for: Weighted gene coexpression correlation network analysis reveals a potential molecular regulatory mechanism of anthocyanin accumulation under different storage temperatures in ‘Friar’ plum
Source: BMC Plant Biol. 2021 Dec 6;21:576. doi: 10.1186/s12870-021-03354-2 (PMC8647467; doi:10.1186/s12870-021-03354-2)
Supplement: Supplementary file 1 — Additional file 1: Figure S1. GO enrichment of the genes in turquoise module. Figure S2. KEGG enrichment of the genes in turquoise module. Figure S3. Correlation analysis of expression profiles of anthocyanin biosynthesis related genes, anthocyanin components and quercetin content. Table S1. The primers used in this study. [file 12870_2021_3354_MOESM1_ESM.docx]

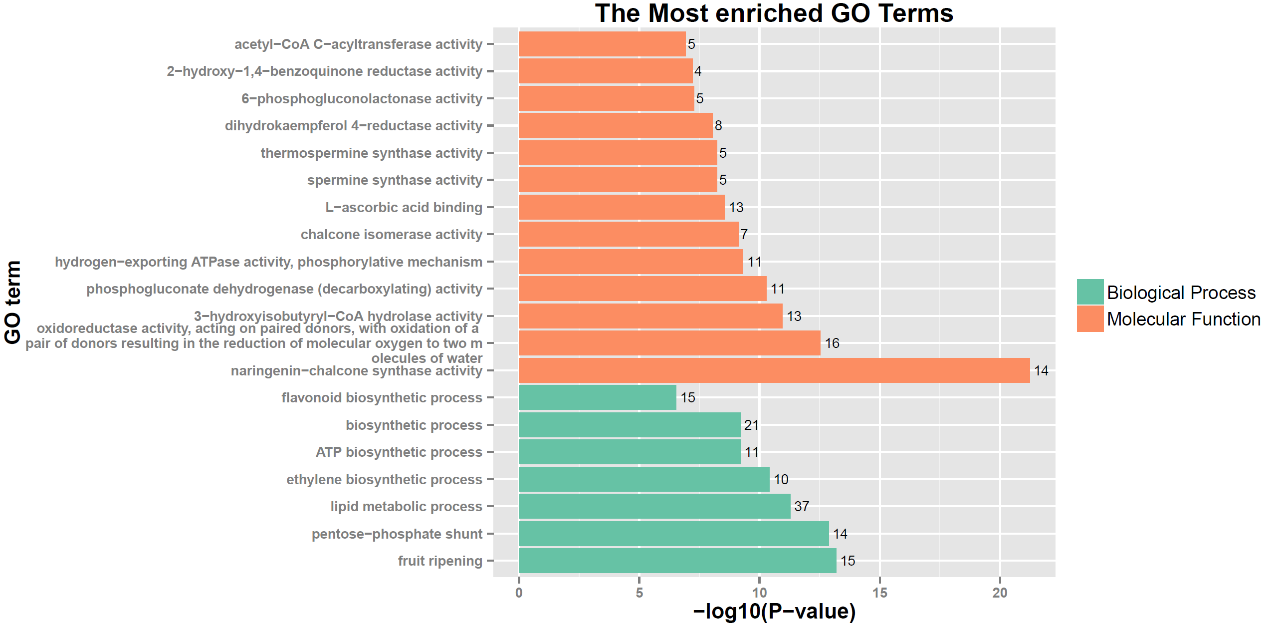


Figure S1. GO enrichment of the genes in turquoise module


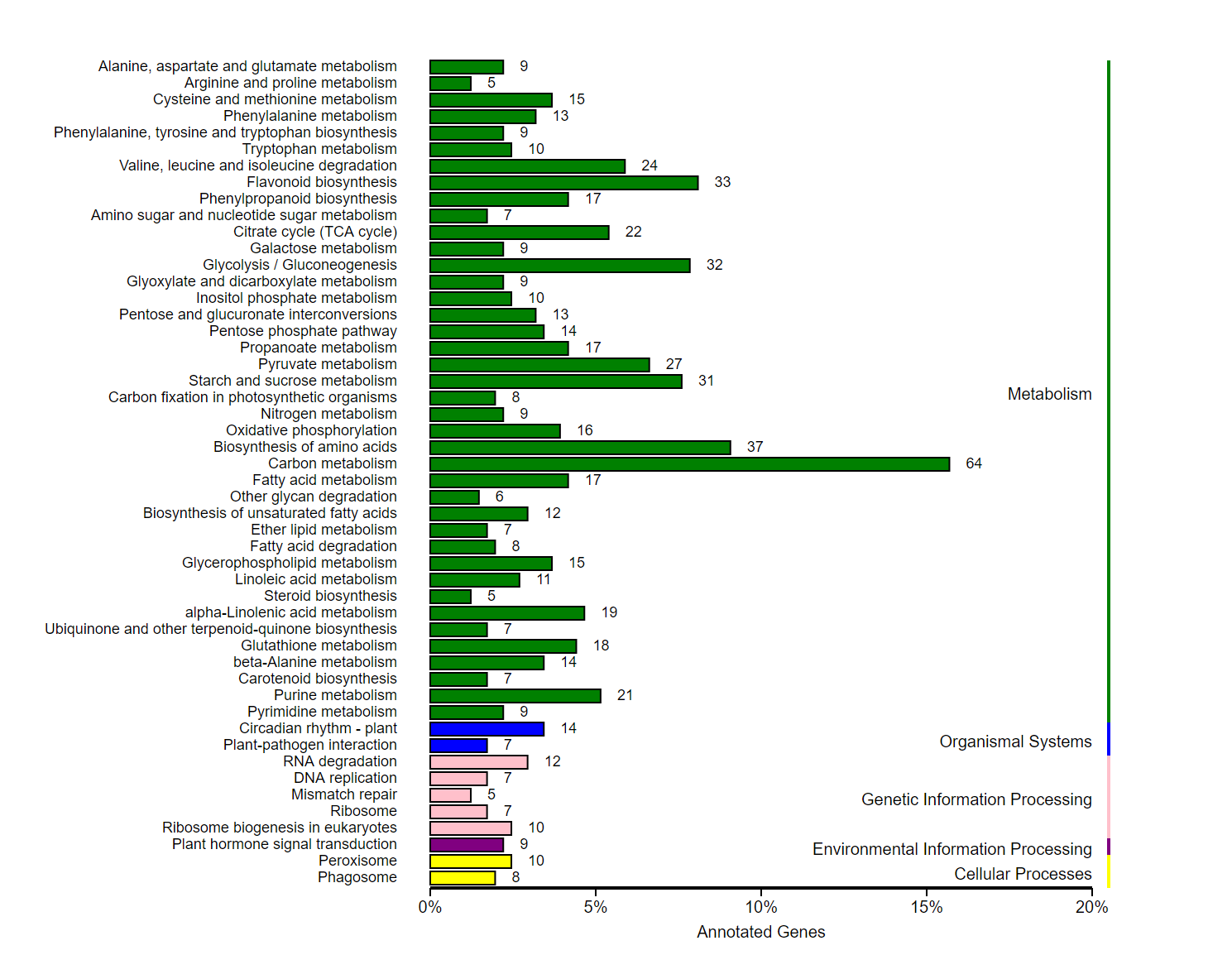


Figure S2 KEGG enrichment of the genes in turquoise module


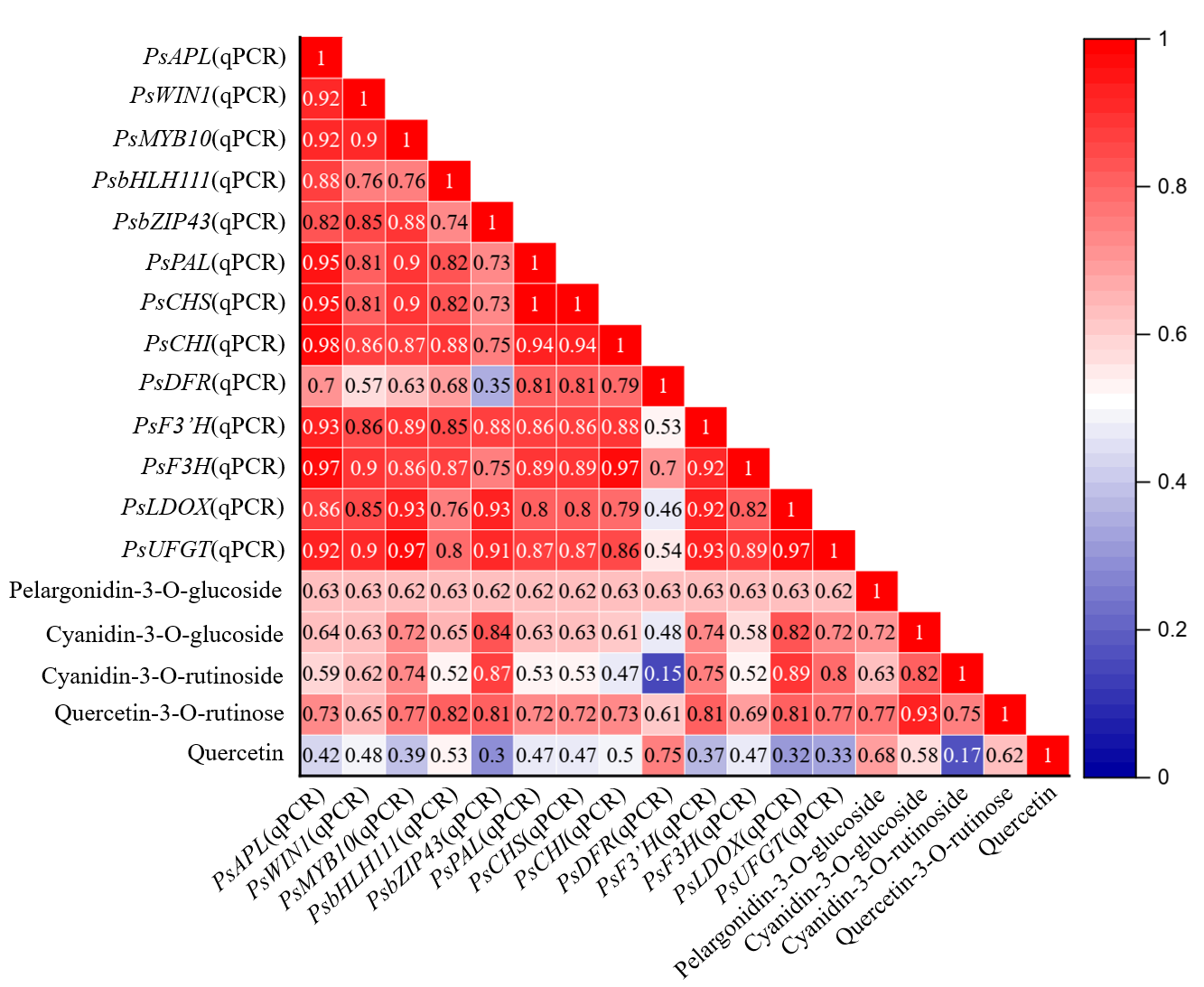


Figure S3 Correlation analysis of expression profiles of anthocyanin biosynthesis related genes, anthocyanin components and quercetin content

(N=13, |r|>0.55 represents a significant correlation between the two sets of data)

Table S1 The primers used in this study

| Gene | Accession no. | Forward | Reverse |
| --- | --- | --- | --- |
| *PsACTQ* | XM_008234719.2 | CTGGACCTTGCTGGTCGT | ATTTCCCGCTCAGCAGTG |
| *PsPALQ* | XM_008245733.2 | AATGCAAGCACTTCAATCTTCC | GAACTCCAGCACTTCCACTATCC |
| *PsCHSQ* | XM_008226357.2 | AGAGTCCAAATTAGCCCTGAAGC | AATAAATAGCACACAGGCACTGG |
| *PsCHIQ* | XM_008235310.2 | CTCGCCGTTAAGTGGAAGG | CTGTGTGAATTTCTCAAATGGACC |
| *PsDFRQ* | XM_007222255.2 | GCCAACAATAAATGGGGTGC | ATTCACGGTTCCTGCTGAGG |
| *PsF3'HQ* | XM_008241809.1 | GAGAAGCCCCATGTCGATGT | GCCAAGGTCCAGTCAAATGC |
| *PsF3HQ* | XM_007202045.2 | GCACAATTACCCTTTTGCTCC | CCCATTGCTCAGAAAATGACC |
| *PsLDOXQ* | XM_007210458.2 | ATTTGGCCTCAAACACCTGC | ATCCCAACCCAAGTGACAGC |
| *PsUFGTQ* | XM_021949698.1 | ATGTCGGACCTTTCAACCTAGC | AGGACAGGCAACCAGTAACGTC |
| *PsAPLQ* | XM_008226275.2 | AGCAACCTCATCCAGAGTGATT | TTGAAGACGACGCTGAACCT |
| *PsWIN1Q* | XM_007216691.2 | ACCCCAGCCAGAAACTAGCA | TGCAACCCTTTCCTCGTCAT |
| *PsMYB10Q* | Pd.00g623010 | AATAAGACCTCAACCCCGAAGC | TTCCCACCAATCCTTTCCGTTT |
| *PsbZIP43Q* | XM_008241838.1 | TGCCCCACTAGGTTCTTCCC | GCTTTGCTCCCCTGCTTCAT |
| *PsbHLH111Q* | Pd.00g1033540 | AAGTTGCATGCGCCTGTGAG | AGCAGATTCTTGCCATAGGGG |
